# Extending the use of the conceptions of learning and teaching (COLT) instrument to the postgraduate setting

**DOI:** 10.1186/s12909-020-02461-2

**Published:** 2021-01-07

**Authors:** Jaime L. Pacifico, Walther van Mook, Jeroen Donkers, Johanna C. G. Jacobs, Cees van der Vleuten, Sylvia Heeneman

**Affiliations:** 1 De La Salle University College of Medicine, De La Salle Medical Health Sciences Institute, Dasmarinas, Cavite, Philippines; 2 Maastricht University Medical Centre, Maastricht, Netherlands; 3 University Medical Centre, Utrecht, Netherlands

## Abstract

**Background:**

Several studies have shown that conceptions of teachers on teaching and learning can influence the teaching practices and behavior in higher education. This association is also found in undergraduate medical education but not yet established in postgraduate medical setting. An instrument, Conceptions of Learning and Teaching (COLT) was developed to measure conception of teachers in undergraduate medical education. COLT is a 3-factor 18-item questionnaire. The objective of this study is to evaluate if COLT is valid for postgraduate medical education.

**Methods:**

We invited postgraduate clinical faculty from 3 hospitals in the Netherlands to fill out the COLT. Confirmatory and exploratory factor analysis were performed to evaluate the fit of the postgraduate clinical faculty data to the COLT. Analysis of variance was done to evaluate if there was difference among the 3 hospitals in terms of the response by the clinical faculty.

**Results:**

Confirmatory factor analysis showed that the postgraduate faculty data had a 2 factor structure after removal of five items. These factors were Teacher Centeredness (TC) and combined Appreciation of Active Learning and Orientation to Professional Practice (A-P) and were considered as comparable to the factors in the original COLT, expressing the post-graduate learning and teaching setting. As several items were removed, the fit was suboptimal, yet did suggest validity for use of the COLT for postgraduate medical education.

**Conclusion:**

The modified COLT can be used to measure conceptions of teaching and learning in postgraduate medical education. We recommend further study to improve the factor structure of the modified COLT.

**Supplementary Information:**

The online version contains supplementary material available at 10.1186/s12909-020-02461-2.

## Background

Several studies have shown that conceptions can influence the teaching practices and behavior in the classroom [1–4]. Many educational researchers have adapted the definition by Pratt [5] that “conceptions are specific meanings attached to phenomena which then mediate our response to situations involving those phenomena … in effect we view the world through the lenses of our conceptions, interpreting and acting in accordance with our understanding of the world … ..our conceptions significantly influence our perception and interpretation of events, people and phenomena surrounding us” [p. 204]. Pratt [5] posits that this relation between conceptions and behavior impacts on how educators think about teaching. Additionally, innovations in teaching and learning are unlikely to happen without concomitant changes in the conception of teaching of the faculty and staff [6, 7]. Kember [6] in his review of teaching conceptions of university academics stated that teaching conceptions are related to measures of quality of learning, and also impact teaching approaches which in turn affect the learning outcomes and how student learn. Kember and Kwan [7] in a study of 17 lecturers in a university about conceptions of teaching, noted that teachers who conceive of teaching as transferring knowledge were more likely to utilize content-centered approaches to teaching, whereas those whose conception of teaching is being facilitators are inclined to utilize learning-centered approaches. These above mentioned studies clearly illustrate a relationship between conceptions of teaching and approaches to teaching and learning. Devlin, however, challenged the associations between conceptions and methods of teaching and the purported relationship between conceptions and improvement on teaching [8]. In their study, Peeraer et al. [9] were not able to confirm a relationship between conceptions of learning and approaches to teaching. These statements, which are contrary to the prevailing notion on the role of conception in teaching, underscore the need for further research regarding conceptions of teaching and its influence on faculty and staff and learning outcomes.

In undergraduate medical education, it was recognized that conceptions of teaching and learning of faculty can promote reflections of teachers and stimulate changes in the teaching behavior [10]. However, clinical teachers rarely receive formal instruction in teaching. Moreover, in medical education, expertise in a particular field is considered equivalent to being a qualified clinical teacher [11, 12]. There is a scarcity of research regarding conceptions of teaching of clinical faculty, in spite of the potential relation between teaching beliefs and behavior of clinical teachers [12, 13]. Since in most institutions the faculty involved in postgraduate education are also involved in undergraduate medical education, it is tempting to assume that the conceptions of undergraduate medical faculty who are also involved in post-graduate education are similar. However, the contexts of undergraduate and postgraduate learning and teaching are different. Many studies on conceptions of learning and teaching are done in secondary and higher education and recently in undergraduate medical education, yet little is done in the postgraduate medical setting. Studying conceptions of teaching in postgraduate medical education is more complicated since a significant part of medical education takes place in clinical settings, presenting with unique challenges to teaching and learning and the circumstances are such that educators cannot control the cases one will encounter and trainees have different levels of competence [12]. Jacobs et al. [10] put forward an instrument, the COLT, that measures conceptions of learning and teaching among undergraduate faculty in two academic institutions for undergraduate medical education in the Netherlands. COLT is a 3 factor, 18 item instrument, the 3 factors are Teacher Centeredness, Appreciation of Active Learning and Orientation to Professional Practice. Using this instrument, they were able to show differences in the conceptions of the faculty between an institution which has a student-centered orientation and an institution which has been using a teacher-centered curriculum [10]. COLT, by design, has neither been used nor shown to be valid in postgraduate education. The objective of this study is to investigate whether the COLT, designed for undergraduate medical education is valid in postgraduate medical education.

## Methods

### Setting

We sampled in 3 medical centers in the Netherlands: 1 academic medical center or hospital (denominated as center 1) and two generic teaching medical centers (denominated as centers 2 and 3). The academic medical center has an established tradition of student-centered education and problem-based learning approach.

### Data collection

#### Instrument

Since COLT was originally designed for undergraduate medical institutions [10] with student-centered curricula, we invited four physicians involved both in undergraduate medical education and postgraduate training to evaluate the original COLT to make it more appropriate in the context of a postgraduate environment. Minor revisions were done and it was agreed that the content of COLT was applicable to the setting of postgraduate residency training. These revisions included replacing the word “students” in the original COLT to “residents” and modifying item number 1 which originally was phrased as “Students should first master basic science knowledge before they can formulate their own goals” to “Residents should first master general medical principles before they can formulate their own learning goals”. COLT is an 18-item 3-factor questionnaire with a five-point Likert scale (1=strongly disagree, and 5=strongly agree). See supplement Table 1.

#### Participants

The COLT questionnaire was distributed to the physician staff of the 3 hospitals. The e-mail invitation was sent via the overarching secretaries of staff at each of the 3 hospitals, the e-mail contained information on the research, informed consent and a link to the questionnaire. One reminder was sent after 4 weeks.

### Data analysis

We performed a confirmatory factor analysis (CFA) and exploratory factor analysis (EFA) to determine if the postgraduate data fitted the 3-factor structure of the original 18-item COLT [10]. We used the following criteria and associated pre-determined cut-off values to gauge goodness of fit: Tucker-Lewis index (TLI > 0.9), comparative fit index (CFI > 0.9), root mean square error of approximation (RMSEA < 0.08) and standardized root mean square residual (SRMR < 0.08). In the present study, a sufficient fit was deemed to have been achieved when 4 criteria produced significant results. Analysis of variance (Anova) and multivariate analysis of variance (Manova) were done to see if there was a significant difference in the scores on the 2 factors among the participants from the three hospitals.

## Results

There were 417 participants in total, 245 male (58.8%) and 170 female (40.8%) while 2 participants did not indicate their gender. Ninety eight percent belonged to age group 30–60 years, as to number of years involved in clinical training 25.7% have been involved for < than 5 years, 28.1% for 5–10 years, and 46% for more than 10 years. There were 173 participants from the academic medical center, 142 from center 2 and 102 from center 3.

A CFA showed that the four fit measures (SRMR = 0.053, RMSEA = 0.047, CFI = 0.886 and TLI = 0.864) were just outside the range for adequate fit criteria, as indicated by Jacobs et al. [10]. The CMIN/DF was 2.02, which is within the range (< 3), but *p* < 0.05, suggesting the fit was not optimal**.** As part of the statistical validation of the questionnaire, the overview of possible disturbances, ordered by the importance provided in the CFA analysis was used. Based on this list, a decision was made which items caused the most disturbance. Then the model was recalculated to determine the fit without those items. Exploratory factor analysis (EFA) was done, and a repeat CFA afterwards showed a good fit if 5 items were removed from the original COLT which led to a 2-factor model. It was shown that removal of several items indeed improved the model and led to an acceptable fit.

Items removed were no. 8 in the original COLT, under the factor Teacher-centeredness (TC), item no. 9, under the factor Appreciation of Active Learning and under the factor Orientation to Professional Practice items 15 and 18. The removal of these items led to the proposed 2 factor model TC and combined Appreciation of Active Learning and Orientation to Professional Practice (A-P). The fit of the 2-factor model was better than the 3-factor model. Removing 1 additional item, item 17, under Orientation to professional practice resulted in good fit: SRMR = 0.049, RMSEA = 0.042, CFI = 0.919 and TLI = 0.902, these are well in the range for adequate fit as indicated by Jacobs et al. [2012]. The CMIN/DF is 1.72, which is within the range (< 3), but still *p*< 0.05, suggesting that the fit is not optimal but usable.

There was a preference for the combined factors A-P across all age groups, regardless of gender, duration of teaching and the hospital surveyed. Tables 1 and 2 summarize these data.

**Table 1:** Mean scores and standard deviation (S.D.) for TC and A-P based on gender

	TC	A-P	No.
Female	3.08 ± 1.46	4.07 ± 1.33	170
Male	3.27 ± 1.58	3.97 ± 1.32	245
All	3.22 ± 1.55	4.01 ± 1.33	417

*2 participants did not indicate gender

**Table 2:** Mean scores and standard deviation (S.D.) according to duration of teaching

Time (years)	TC	A-P	No.
< 5	3.20 ± 1.53	4.04 ± 1.34	107
5–10	3.18 ± 1.48	3.96 ± 1.31	117
> 10	3.22 ± 1.59	4.02 ± 1.34	193
All	3.22 ± 1.55	4.01 ± 1.33	417

There was no significant difference in the responses of the participants among the three hospitals surveyed in this study using Anova and Manova. The Cronbach’s alpha overall was 0.477, for TC 0.703 and for A-P 0.504. Supplement Table 2 shows the postgraduate COLT questionnaire, and the overall responses**.**

## Discussion

Although the COLT [10] was designed for student-centered medical education in an undergraduate setting, this study showed the questionnaire is applicable in postgraduate medical education, although with some modifications. The postgraduate COLT met the criteria for goodness of fit although the fit is not optimal. Our study also revealed that among the 417 physicians who participated there was a preponderant preference for the items originally included in the factors “appreciation of active learning” and “orientation to professional practice” which were combined into a single factor after the CFA. In the original COLT [10] both these factors exemplified concepts consistent with student-centered education in a higher education setting. Most of the instruments available in the literature to measure conceptions of teaching and learning are designed for higher education and undergraduate medical education.

Postgraduate medical training, in contrast with undergraduate medical education, is faced with distinct challenges in the context of daily clinical care. Both clinical teachers and residents have to combine the responsibilities of daily clinical care with learning and learning opportunities. The trainees have limited options to choose and select and therefore must deal with the available cases as learning opportunities. Likewise, the faculty has to adjust to the cases to teach or discuss. Postgraduate trainees in the setting of work-based learning have dual roles as learners and as ‘employees’ wherein they are also expected to provide service to their patients [14]. In addition, in postgraduate training, the educational alliance between trainee and clinical teacher takes a next level, as the trainee is also a junior-colleague and responsible member of the team [15, 16]. These contextual factors affect the learning environment [12, 17]. The trainees are expected to cope with the limitations presented by the above mentioned conditions, and try to minimize the distractions these can have on their training**.**

Our study showed some important differences in the factor structure of the COLT in post-graduate settings in contrast with the original COLT, which was intended for undergraduate medical education. In the postgraduate COLT, using statistical analysis 5 items were removed to improve the fit. These differences were anticipated, given that these could be related to the specific context of workplace-based teaching and learning in postgraduate training as delineated in the previous paragraph. For the separate items, some additional remarks can be made:

For the item: “*When residents collaborate they teach each other the wrong things”*, this item was part of the teacher-centeredness factor in the undergraduate COLT but has to be removed in the current study to improve the fit. We speculate that physicians who participated in the study most probably felt that at the level of postgraduate setting, residents cannot afford to make mistakes or teach each other ‘wrong things’ since they are involved in the management of patients already, which would make the item irrelevant.

For the item: *“Residents learn a great deal by explaining the subject matter to each other.”* This item may have given the impression that in a postgraduate setting theoretical learning is still dominant which is opposite the actual condition, where decisions made can impact a person’s life or quality of life. Sociocultural theories are increasingly used in graduate medical education to explain how learning occurs, this includes the theory of situated learning. Situated learning emphasizes that learning is intertwined with the context, the social relations and practices [18–20]. Lave and Wenger [18] expanded this to legitimate peripheral participation as a process by which trainees start as unrecognized members to being respected members of communities of practice.

For the item: “*Being introduced to the day-to-day practice of their future profession motivates residents to learn”.* Although this item fits the student-centered education of center number 1 in undergraduate medical education, such may not be the case in postgraduate educational setting and this may be less evident in the workplace learning where clinical duties prevail. In addition, the residents are already working in their ‘future’ profession, so this may have given a variety of responses that did not lead to a fit.

For the item: *“I think that interactions between me and the residents are an important aspect of my teaching”.* The removal of this item was not expected, in a study done among residents involving internal medicine and pediatric residents, a strong motivator to learning for the residents was the engagement between staff and residents [21]. A similar finding although involving undergraduate medical education but done in the same cultural context as our current study is reported by Strand et al. [22]. They described learning as partnership, as one of the main three main themes, where the interaction with the supervisor plays a significant role, and in addition the supervisory relationship gave way to reciprocal learning [22]. The fact that the responses of the physicians surveyed did not support this item could reflect an ongoing dichotomy between the perceptions of staff and perception of the trainees. In our view this might require further investigation, in the light of the above studies which strongly support that interaction between trainees and supervisors play a crucial role and promote learning among undergraduate and postgraduate medical trainees.

For the item: *“Discussing topics with each other helps residents learn how to deal with different points of view, so as to gain a deeper understanding”*, Teunissen et al. [23] proposed a framework on how residents learn which postulates that the starting point of learning is getting involved in work-related activities. For residents the ‘experience‘initiates the construction of meaning which generates personal knowledge [23]. This is similar to experiential learning theory which highlights that trainees construct knowledge and meaning based on their real-life experiences [24]. Thus, for the residents the emphasis at this point is on actual activities that induce learning and not on theoretical discussion.

The principal advantages and benefits of measuring the conceptions of the faculty were found to include evaluation and improvement of teaching, faculty development and peer reviews of teaching [25]. This is echoed by the study of Strand et al. [22] who investigated the physicians’ conception focusing on learning in the clinical workplace and how clinical supervisors contribute to learning. One of their conclusions was that mapping the workplace supervisor conceptions of learning can be a valuable starting point for medical schools and educational developers working with changes in clinical educational and faculty development practices.

In the postgraduate setting, Calkins et al. [26] believe that helping the faculty to develop a complex understanding of teaching is a fundamental component in a faculty member’s professional development. Perspectives on teaching are philosophical orientations to knowledge, learning and the responsibility of an educator [27] and healthy discussion on a teacher’s perspectives is a good way to understand philosophies their colleagues hold onto [28].

In this study, an existing instrument was used as the starting point, which has certain advantages which includes time and the availability of a preexisting construct. We agree that further study and validation are needed to come to an instrument to measure conception among postgraduate clinical teachers*.* We recommend future studies that can improve the reliability of the current postgraduate COLT, possibly with additional items tailored specifically to workplace learning in the postgraduate setting. We consider it a limitation of our study that although we found a new factor structure, the factors are not optimally represented by the items. Given the deletion of items, a smaller number of items remained, also explaining the suboptimal Cronbach’s alpha overall and of the two factors.

## Conclusion

There are established benefits in being able to measure the conceptions of teaching and learning, however in postgraduate medical education there is a lack of instruments that can be used to measure faculty conceptions. The postgraduate COLT, although the psychometric properties are not optimal, may be used for this purpose with the limitations presented. For clinical teachers, becoming aware of their conceptions can be a stepping stone to improve their teaching approaches, benefitting not only the medical trainees but the patients as well.

## Supplementary Information

**Additional file 1: Table S1.** Original COLT Questionnaire. **Table S2.** Postgraduate COLT Questionnaire and overall scores.

## Data Availability

The datasets used and/or analyzed during the current study are available from the corresponding author on reasonable request.

## Abbreviations

COLT: Conceptions of learning and teaching
TC: Teacher centeredness
A-P: Appreciation of active learning-Orientation to professional practice
CFA: Confirmatory factor analysis
EFA: Exploratory factor analysis
TLI: Tucker-Lewis index
CFI: Comparative fit index
RMSEA: Root mean square error of approximation
SRMR: Standardized root mean square residual
Anova: Analysis of variance
Manova: Multivariate analysis of variance
SD: Standard deviation
