# Supplementary material for: Extending the use of the conceptions of learning and teaching (COLT) instrument to the postgraduate setting
Source: BMC Med Educ. 2021 Jan 7;21:32. doi: 10.1186/s12909-020-02461-2 (PMC7792289; doi:10.1186/s12909-020-02461-2)
Supplement: Supplementary file 1 — Additional file 1: Table S1. Original COLT Questionnaire. Table S2. Postgraduate COLT Questionnaire and overall scores. [file 12909_2020_2461_MOESM1_ESM.docx]

**Supplement Table 1. Original COLT Questionnaire**

| **Factor 1: Teacher centeredness** | |
| --- | --- |
| 1. | Residents should first master general medical principles before they can formulate their own learning goals. |
| 2. | I think that in small group learning, the clinical faculty determines what the residents should learn instead of the residents determining their own learning goals*.* |
| 3. | Residents learn best when the learning process is guided by an expert who has an overview of the field of interest. |
| 4. | When residents discuss a topic without an expert being present, they do not know at the end of the session if the questions have been answered correctly. |
| 5. | There is a logical sequence to learning. |
| 6. | As a teacher I have to indicate clearly what is important and what is less important for the residents to know. |
| 7. | I think that as an expert in my field I am eminently suitable to transmit my knowledge to residents and that residents should not have to look up that knowledge for themselves. |
| 8. | When residents collaborate they teach each other the wrong things. |
| **Factor 2. Appreciation of Active Learning** | |
| 9. | Residents learn a great deal by explaining the subject matter to each other. |
| 10. | Learning materials and teaching should invite residents to come up with examples to illustrate the subject matter. |
| 11. | Small group learning motivates residents to study*.* |
| 12. | I think it is more important for residents to be able to analyze and critically appraise  the subject matter than to memorize facts. |
| 13. | I think it is important that residents advise each other about the best ways to study. |
| **Factor 3: Orientation to Professional Practice** | |
| 14. | I think it is important that educational assignments are derived as much as possible from the residents’ future professional practice |
| 15. | Being introduced to the day-to-day practice of their future profession motivates residents to learn. |
| 16. | It is good learning outcome when residents demonstrate that they can apply their  knowledge during their activities in situations in professional practice. |
| 17. | I think that interactions between me and the residents are an important aspect of my teaching. |
| 18. | Discussing topics with each other helps residents learn how to deal with different points of view, so as to gain a deeper understanding. |

**Supplement Table 2. Postgraduate COLT Questionnaire and overall scores**

|  | ***Item*** | ***Overall Score***  ***(mean ± S.D.)*** |
| --- | --- | --- |
|  | ***Factor 1. Teacher – Centeredness*** |  |
| 1. | Residents should first master general medical principles before they can formulate their own learning goals. | 3.06 ± 1.11 |
| 2. | I think that in small group learning, the clinical faculty determines what the residents should learn instead of the residents determining their own learning goals | 2.85 ± 0.85 |
| 3. | Residents learn best when the learning process is guided by an expert who has an overview of the field of interest. | 3.58 ± 0.83 |
| 4. | When residents discuss a topic without an expert being present, they do not know at the end of the session if the questions have been answered correctly. | 3.34 ± 0.93 |
| 5. | There is a logical sequence to learning | 3.33 ± 0.93 |
| 6. | As a teacher I have to indicate clearly what is important and what is less important for the residents to know. | 3.45 ± 0.85 |
| 7. | I think that as an expert in my field I am eminently suitable to transmit my knowledge to residents and that residents should not have to look up that knowledge for themselves. 2.815 0.8559 | 2.82 ± 0.86 |
|  | ***Factor 2. Active Learning/ Orientation to Professional Practice*** |  |
| 8. | Learning materials and teaching should invite residents to come up with examples to illustrate the subject matter. | 3.93 ± 0.65 |
| 9. | Small group learning motivates residents to study. | 4.05 ± 0.62 |
| 10. | I think it is more important for residents to be able to analyze and critically appraise the subject matter than to memorize facts. | 3.62 ± 0.92 |
| 11. | I think it is important that residents advise each other about the best ways to study. | 3.69 ± 0.67 |
| 12. | I think it is important that educational assignments are derived as much as possible from the residents’ future professional practice. | 4.03 ± 0.63 |
| 13. | It is good learning outcome when residents demonstrate that they can apply their knowledge during their activities in situations in professional practice. | 4.35 ± 0.52 |
